# Recovery of daily-life walking after total knee arthroplasty: A two-year longitudinal study and comparison with healthy controls

**DOI:** 10.1016/j.ocarto.2026.100835

**Published:** 2026-06-04

**Authors:** F.J. Bruning, R.J. Boekesteijn, K.C. Defoort, S. Susan, C.H.M. van den Ende, K. Smulders

**Affiliations:** a Department of Research, Sint Maartenskliniek, Nijmegen, the Netherlands; b Department of Orthopedic Surgery, Sint Maartenskliniek, Nijmegen, the Netherlands; c Department of Rheumatology, Radboudumc, Nijmegen, the Netherlands

**Keywords:** Total knee arthroplasty, Daily-life walking, Wearable sensors, Real-world gait

## Abstract

**Objective:**

Total knee arthroplasty (TKA) aims to improve walking in individuals with end-stage knee osteoarthritis, but the extent of daily-life walking recovery remains unclear. This study assessed whether individuals regain daily-life walking comparable to healthy people one year after surgery and examined recovery over two years.

**Method:**

This longitudinal study assessed daily-life walking with inertial sensors before, and one and two years after TKA. Thirty-one individuals with unilateral knee osteoarthritis were compared to thirty-one healthy participants without joint pain. The primary outcome was daily-life gait speed at one year. Differences between the TKA group and healthy participants at one year were assessed with t-tests. Pre-to-post-TKA comparisons were analyzed using linear mixed models.

**Results:**

One year after surgery, gait speed of the TKA group was 0.10 m/s lower compared to healthy participants. From preoperatively to one year postoperatively, gait speed increased by 0.10 m/s (95% CI: 0.04 to 0.15), with 12 out of 27 individuals improving beyond the threshold of ≥0.1 m/s change. Stride length and stride time improved at one year post-surgery, but the maximum gait bout length did not improve. No improvements were observed from 1 to 2 years postoperatively.

**Conclusion:**

One year after TKA, individuals exhibited only minor daily-life walking limitations compared with healthy peers. Although walking parameters improved relative to preoperative values, these changes were modest and of questionable clinical relevance, likely reflecting the relatively high functional status of patients before surgery.

## 1 Introduction

Individuals with end-stage knee osteoarthritis (OA) experience major limitations in daily mobility due to pain [1]. For these individuals, total knee arthroplasty (TKA) is a treatment option to improve daily mobility by reducing pain and improving knee function [2]. In a previous study from our group, we observed that individuals scheduled for TKA exhibited clear impairments in daily-life walking when compared to healthy older adults [3]. Unsurprisingly, enhancing daily-life walking is a primary treatment goal for individuals undergoing TKA [[4], [5], [6]], and a key determinant of treatment satisfaction after TKA [7]. Despite its clear importance, recovery of walking after TKA is still primarily evaluated through self-reported measures, with only scarce use of objective assessments.

Multiple studies have underscored a complementary role of objective walking assessment to self-reported outcomes for characterizing functional abilities after TKA [[8], [9], [10], [11], [12], [13]]. However, a key limitation of clinical walking assessment is its low ecological validity, as it measures capacity (i.e. what someone can do in a controlled setting) rather than performance (i.e. what someone does in their own environment) [14,15]. Hence, monitoring daily-life walking may offer a more accurate understanding of functional recovery over time following TKA [16]. To capture performance, wearable inertial movement sensors can be used, capturing walking data continuously and remotely [16].

Although self-reported outcomes typically indicate substantial recovery of physical function one year post-TKA [10,11,17,18], it remains unclear to what level objectively measured walking in daily life improves. To date, only a few studies have evaluated changes in daily-life walking metrics after TKA [19,20]. One study assessed walking metrics at 3 months post-TKA and found no improvements, likely due to the early stage of rehabilitation [19]. Another study examined recovery at one year post-TKA and observed improved knee flexion during walking [20]. Longer term evaluation of daily-life walking, including spatiotemporal gait metrics to address walking performance recovery, has not been done. Therefore, the aims of this study were to compare daily-life walking metrics of individuals one year following TKA to an age- and sex-matched healthy cohort, and to evaluate the recovery of daily-life walking up to two years post-TKA. Based on results obtained during gait assessments in the clinical setting [11], we hypothesized that walking metrics would improve after TKA, but would remain impaired compared to healthy participants.

## 2 Methods

### 2.1 Participants

All individuals who were candidates for posterior cruciate-retaining TKA at the Sint Maartenskliniek between July 2020 and December 2022 were screened for eligibility by a research nurse. Individuals were eligible to participate in this study if they had unilateral non-inflammatory knee OA with an indication for TKA, and had less than moderate to severe pain in the hips, ankles or the contralateral knee (average score >4 on items 3–6 of the Brief Pain Inventory – Short Form [21]). Furthermore, individuals with previous knee, hip or ankle replacement surgery, or any other musculoskeletal or neurological diseases affecting walking or balance were not eligible for participation. Healthy participants were recruited from the community through public advertising and were individually matched to the individuals undergoing TKA based on age (maximum difference of 5 years) and sex. Healthy participants were eligible if they were in stable health (classification of 3 or less on the American Society of Anesthesiologists Physical Status Classification System [22]), had no previous or scheduled joint replacement surgery, and had less than moderate to severe pain in the knee, hip, or ankle joints (defined by an average score >4 on items 3–6 of the Brief Pain Inventory – Short Form [21]). The complete list of inclusion and exclusion criteria for both individuals undergoing TKA and healthy participants is provided in Appendix A.

This study was powered with gait speed as primary outcome measure, aiming to detect differences between healthy participants and individuals at one year post-TKA with sufficient precision. We considered a confidence interval half-width of 0.10 m/s informative, because this value corresponds to the lower boundary of the most robust reported values for clinically important change in gait speed established in other populations (0.10–0.17 m/s) [23] and has been associated with important health outcomes and physical functioning in older adults [24,25]. To detect a difference of 0.17 m/s [26] between healthy participants and individuals one year post-TKA, with a confidence interval half-width of 0.10 m/s, 29 participants were required. Taking into account a potential 10% dropout rate, 32 individuals were required for each group (TKA and healthy subjects).

Written informed consent was obtained from all participants prior to participating in this study. Medical-ethical approval was obtained from CMO Arnhem-Nijmegen (2019–5824), and all study procedures were in accordance with the Declaration of Helsinki. This study was pre-registered at Open Science Framework (https://osf.io/64ejm). The STROBE guidelines [27] were used to report this study.

### 2.2 Data collection

Individuals undergoing TKA were assessed up to two months before TKA (baseline), and one year (range: 11–13 months) and two years (range: 22–26 months) post-TKA, whereas healthy participants were assessed at a single time point. Baseline anthropometric measures (sex, age, height, body mass) were obtained for all participants. Before TKA, anterior-posterior radiographic images of the knee were obtained for the TKA group and graded for OA severity using the Kellgren-Lawrence grading system [28] by an experienced orthopedic surgeon.

**Fig. 1:**
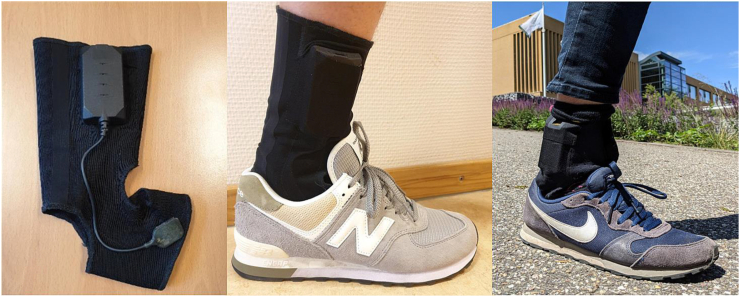
Wearable sensors embedded in socks. Upper part of the sensor (left panel) is the battery and memory drive, connected to the sensor on the dorsum of the foot.

At each timepoint, daily-life walking was monitored using three inertial movement units (IMUs; APDM Wearable Technologies, Portland, OR, United States of America) which recorded data at a sample frequency of 128 Hz. Two sensors were embedded in socks and positioned on the dorsum of each foot and one sensor was worn on the lower back at the sacrolumbar level using a waistband (Fig. 1). Participants were asked to wear the sensors for 5–7 consecutive days and were instructed to start wearing the sensors in the morning when they started their daily activities. The sensors had a battery life of 10–12 h and were charged overnight. Data were stored locally on the sensors, and sensors were returned to the researchers via parcel service.

Pain during rest and activity over the preceding week was reported using a numeric rating scale (NRS, 0 (no pain) – 10 (worst pain)) at each assessment. Self-reported physical function of individuals in the TKA group was assessed using the Knee Injury and Osteoarthritis Outcome Score – Physical Function shortform (KOOS-PS, 0–100), with lower scores indicating better physical function [29]. All questionnaires were distributed and completed online through CastorEDC at each timepoint. At 3 months post-TKA, participants reported the total number of completed physical therapy sessions.

### 2.3 Surgical procedure

All individuals scheduled for TKA received the Journey II Cruciate Retaining knee system (Smith & Nephew, Memphis, TN, USA) with patellar button. TKA was performed by one of three specialized orthopedic surgeons, using a medial parapatellar approach and a mechanical alignment technique. After TKA, all patients followed a rehabilitation protocol with mobilization on the day of surgery and hospital discharge within two days. After hospital discharge, patients were referred to outpatient physical therapy.

### 2.4 Data processing

Raw sensor data were processed using the algorithm of Shah et al. (2020) to extract the episodes in which a participant was walking (i.e., ‘gait bouts’) [30]. Both the study hardware and processing software were provided by APDM (Portland, United States of America), and the gait bout detection pipeline was developed for this specific sensor type and configuration [30]. Gait bouts consisting of ≥4 consecutive strides were processed using the Mobility Lab algorithm (APDM, Portland, United States of America [31]) to calculate gait speed, stride time and stride length per stride. Turns during walking were identified as described in El Gohary et al. (2013) [32]. For each turn during walking, the maximum turning velocity was calculated if the turn was larger than 45° and lasted between 0.5 and 10 s. Initial sanity checks were performed on a subset of the baseline data to verify gait bouts identified by the algorithm aligned with plausible walking patterns.

**Fig. 2:**
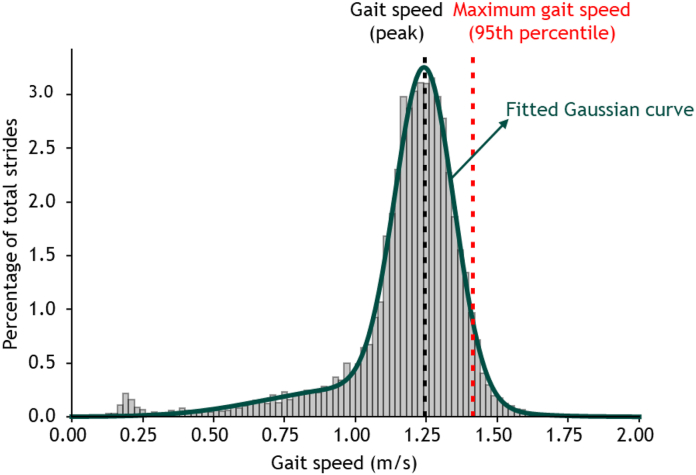
**–** Example of a gait speed distribution of all strides of one individual at one time point. The green curve represents the Gaussian curve fitted over the distribution. The vertical black dotted line represents gait speed, defined as the peak of the distribution. The vertical red dotted line represents the maximum gait speed, defined as the 95th percentile of the distribution.

The primary outcome of this study was gait speed, which was determined from each participant’s stride data. For each participant at each time point, a histogram of all strides was constructed, and Gaussian curves were fitted to the resulting distribution. If the distribution was unimodal, the single peak was taken as gait speed (Fig. 2). When the fitted distribution showed two distinct peaks, gait speed was defined by the dominant peak (i.e. most common gait speed). In these cases, the dominant peak corresponded to the peak at the higher gait speed, which was considered most representative of habitual gait speed, because lower gait speeds are more likely to reflect short, variable gait bouts [14,33]. Furthermore, the maximum gait speed was determined from the frequency distribution, as the 95th percentile value of the distribution (Fig. 2). Due to non-normally distributed data, the median stride time and stride length were calculated over all strides per participant. The maximum gait bout length and the mean number of strides per monitored hour were calculated as measures of walking activity. Turning performance at each time point was quantified as the median peak turning velocity of all turns for each participant.

In a supplementary analysis we analyzed the association between gait speed and gait bout length, to identify if differences in gait speed could be due to differences in walking activity patterns. This analysis is described in Supplementary Material I.

### 2.5 Statistical analysis

Prior to analysis, all walking metrics were visually inspected for extreme or implausible values. Assumptions of homoscedasticity and normality of residuals were assessed by visual inspection of residual and Q-Q plots. Between-group differences (one year post-TKA vs. healthy participants) in walking metrics at one year post-TKA were first explored using independent t-tests. Because higher BMI has been associated with lower gait speed and altered gait characteristics [34,35] and is a known risk factor for knee OA [36], we specified BMI as a potential confounder a priori in the comparison of post-TKA and healthy participants. BMI was added as a covariate to the linear regression models, with the walking metrics as dependent variable, and group (TKA vs. healthy participants) as independent factor.

Changes in walking metrics between baseline, one, and two years post-TKA were analyzed using linear mixed models, with the respective walking metric as dependent variable, time point as fixed effect and subject ID as random effect. Time point was coded using two dummy variables representing one year and two years post-TKA, with baseline as a reference category. This model outputted an intercept corresponding to the baseline mean, the first coefficient estimating the mean difference between one year and baseline, and the second coefficient estimating the mean difference between two years and one year post-TKA. Means at baseline and mean differences with 95%-confidence intervals (95%CI) were reported for each outcome. Clinical relevance of the change from baseline to one year post-TKA was evaluated against a prespecified threshold of 0.10 m/s for gait speed. As no TKA-specific threshold has been established, we selected the lower end of the MCID range reported in a systematic review of patient groups with different gait impairments [23]. We chose the lower end of this range to reduce the risk of overlooking potentially clinically relevant improvements in gait speed. As a post-hoc analysis, the association between baseline gait speed and the change in gait speed at one year post-TKA was evaluated using Pearson’s correlation coefficient.

For comparisons between the TKA group and healthy participants, individuals with missing data were excluded from the analysis. For longitudinal analyses, linear mixed models included all available observations and handled missing data (i.e. lost to follow-up or no measurements available) under the assumption of missing at random.

Statistical analyses were conducted using the lme4 and stats package in R (version 4.4.0).

## 3 Results

**Fig. 3:**
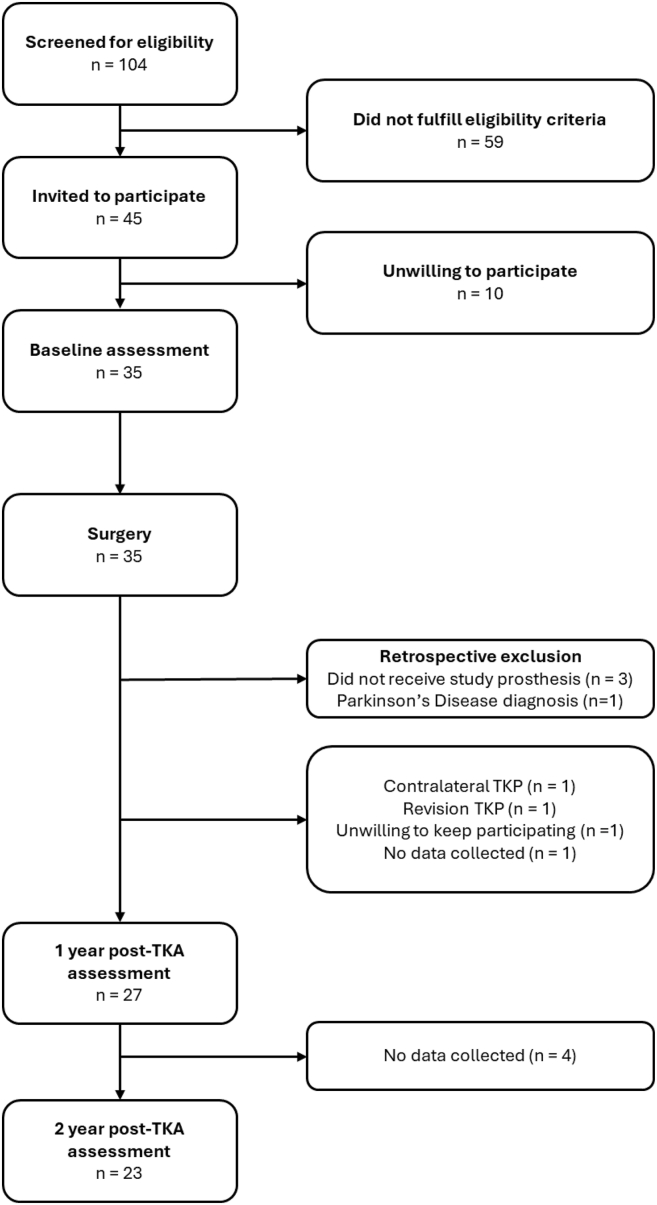
Participant flow throughout the study, showing number of individuals for eligibility assessment, in study, and loss to follow-up. Number of individuals at baseline, one year post-TKA and two years post-TKA represents the number of individuals of whom gait speed (primary outcome) was available.

Of the 104 individuals screened for eligibility, 59 individuals did not meet the eligibility criteria and 10 individuals were not willing to participate (Fig. 3). As a result, 35 individuals were assessed at baseline, from whom 3 participants had to be excluded retrospectively as they did not receive the planned prosthesis. Between surgery and one year post-TKA, 5 individuals dropped out (Fig. 3). Twenty-seven individuals at one year post-TKA and 23 individuals at two years post-TKA had data for gait speed (primary outcome).

Characteristics of the study population at baseline and one year post-TKA, and of the healthy participants are summarized in Table 1. One year post-TKA, participants had a higher BMI compared to the healthy group (mean difference: 4.0 kg/m^2^, 95%CI: [2.3, 5.7]). The TKA group reported a mean of 19 (sd 6) physical therapy sessions in the first 3 months post-TKA. In the TKA group, the IMUs recorded a mean time of 61 (sd 18) hours at baseline, 45 (sd 21) hours one year post-TKA, 51 (sd 33) hours two years post-TKA. Healthy participants recorded 58 (sd 17) hours with IMUs (Table 1).

**Table 1:** Characteristics of the study population.

	Individuals TKA Baseline (n = 31)	Individuals TKA 1 year post-TKA (n = 27)	Healthy participants (n = 27)	Mean difference 1 year post-TKA – Healthy participants [95%CI]
Sex (M:F)	12:19	11:16	11:16	–
Age (years)	65 (10)	65 (9)	64 (10)	1 [−4, 6]
Height (m)	1.73 (0.10)	1.72 (0.10)	1.75 (0.07)	−0.03 [−0.07, 0.02]
Weight (kg)	85.4 (15.0)	84.1 (15.4)	74.1 (11.2)	10.0 [2.8, 17.2]
BMI (kg/m^2^)	28.4 (3.3)	28.1 (3.4)	24.1 (2.8)	4.0 [2.3, 5.7]
Kellgren-Lawrence grade (I:II:III:IV)	0:0:10:21	0:0:9:18	–	–
Physical therapy sessions at 3 months (n)	–	19 (6)	–	–
Recorded time with IMUs (h)	61 (18)	45 (21)	58 (17)	–

Characteristics of the study population at baseline and 1 year post-TKA, and of the healthy participants. Means and standard deviations are shown for age, height, body mass, BMI, number of physical therapy sessions, and recorded time with IMUs. Mean difference and 95%CI between 1 year post-TKA and healthy participants is shown. Count data are shown for sex and Kellgren-Lawrence grade. BMI = Body mass index, 95%CI = 95%- confidence interval.

Comparisons of walking metrics between individuals at one year post-TKA and healthy participants are shown in Table 2. Individuals at one year post-TKA had a lower gait speed (−0.10 m/s, 95%CI: [−0.18, −0.01], *p* = 0.004) and a lower maximum gait speed (−0.11 m/s, 95%CI: [−0.18, −0.03], *p* = 0.001) than healthy participants. Maximum bout length was lower in the group one year post-TKA compared to healthy participants (−244 strides, 95%CI: [−496, −18], *p* = 0.03). Stride time, stride length, turning velocity and strides per hour were comparable between healthy participants and individuals one year post-TKA. When BMI was added to the models, the mean differences changed minimally, but did change statistical significance. After BMI adjustment, the differences in gait speed, maximum gait speed, and maximum bout length were no longer statistically significant, while the difference in stride length became statistically significant (−0.09 m [0.17–0.00] shorter strides in post-TKA group; p = .04).

**Table 2:** Comparison of mobility metrics between one year post-TKA and healthy participants.

	1 year post-TKA n = 27	Healthy participants n = 27	Mean difference [95%CI]	*p-*value	Corrected mean difference [95%CI]	*p-*value
Gait speed (m/s)	1.25 [1.19, 1.31]	1.34 [1.27, 1.41]	−0.10 [−0.18, −0.01]	0.04	−0.09 [−0.20, 0.02]	0.09
Maximum gait speed (m/s)	1.42 [1.37, 1.47]	1.53 [1.47, 1.59]	−0.11 [−0.18, −0.03]	0.01	−0.06 [−0.15, 0.03]	0.17
Stride time (s)	1.13 [1.09, 1.17]	1.10 [1.07, 1.13]	0.04 [−0.01, 0.09]	0.16	0.01 [−0.05, 0.07]	0.64
Stride length (m)	1.34 [1.29, 1.39]	1.41 [1.35, 1.46]	−0.07 [−0.14, 0.00]	0.07	−0.09 [−0.17, −0.00]	0.04
Peak turning velocity (deg/s)∗	96.2 [91.2, 101.1]	102.0 [97.3, 106.6]	−5.8 [−12.4, 0.8]	0.08	−5.0 [−13.0, 3.0]	0.21
Maximum bout length∗∗	391 [233, 549]	635 [476, 793]	−244 [−496, −18]	0.03	−77 [−350, 196]	0.57
# Strides/ hour	237 [172, 301]	271 [202, 340]	−34 [−126, 58]	0.46	−31 [−144, 83]	0.59

Means and 95%CI are shown for mobility metrics one year post-TKA and healthy participants. Mean differences, 95%-Confidence Intervals (95%CI) and *p*-values are shown for the comparison of the one year post-TKA group and healthy participants. Corrections for differences in BMI between groups were conducted and presented as the corrected mean difference. TKA = total knee arthroplasty, ∗ = data based on comparison for n = 25, ∗∗ = data based on comparison for n = 26.

**Fig. 4:**
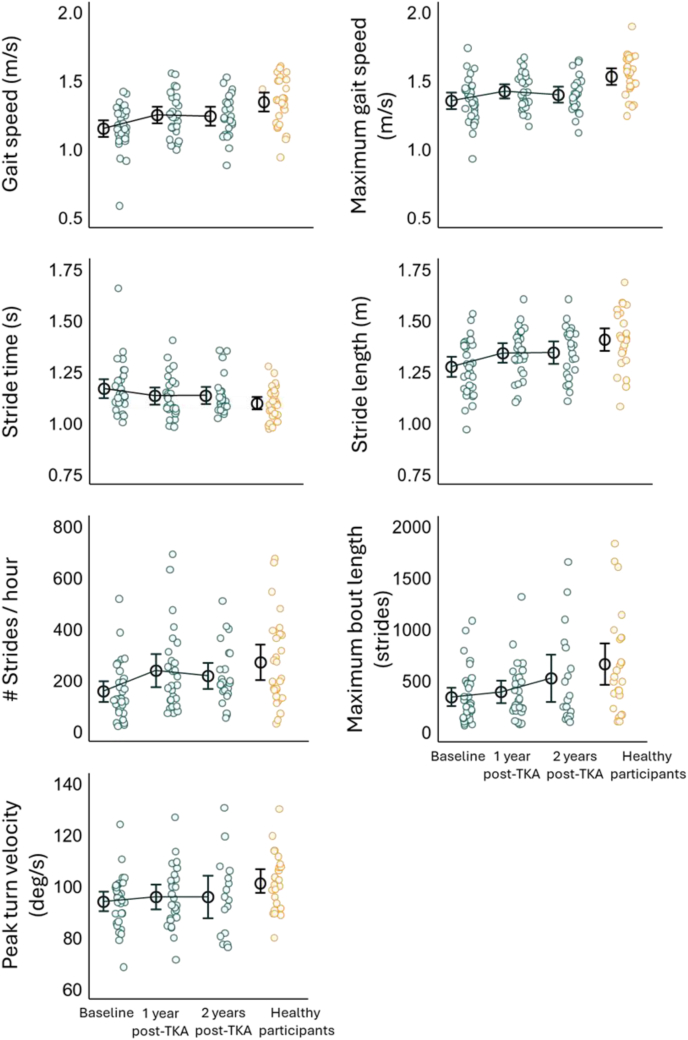
Walking metrics at baseline, one year post-TKA, two years post-TKA and healthy participants. Green (TKA group) and orange (healthy participants) dots represent individual data. White dots represent the mean per group and time, and error bars represent the 95%CI. TKA = total knee arthroplasty.

**Fig. 5:**
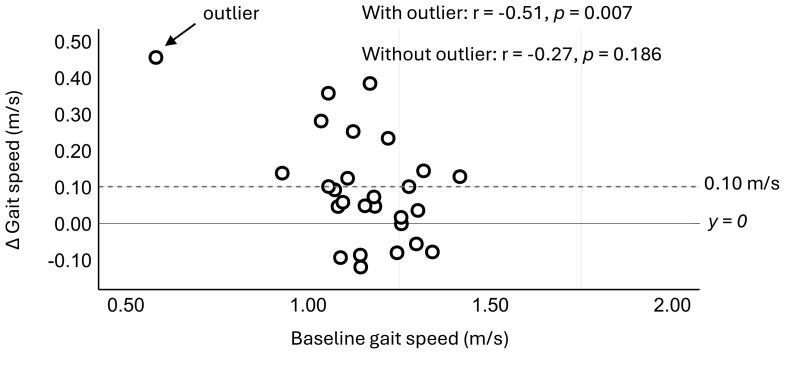
Correlation between baseline gait speed and the difference between one year post-TKA and baseline. Each dot represents one individual. The dotted line represents the threshold for change in gait speed (0.10 m/s).

Changes in daily-life walking metrics from baseline to one and two years post-TKA are shown in Fig. 4 and Table 3. Compared to baseline, one year post-TKA dominant gait speed had increased with 0.10 m/s (95%CI: [0.04, 0.15], *p* = 0.001). Twelve participants out of the 27 TKA participants exceeded the prespecified threshold of ≥0.1 m/s change in gait speed at one year (Fig. 5). Gait speed of one individual had deteriorated by more than the prespecified threshold of 0.10 m/s. A post-hoc exploratory analysis showed that baseline gait speed was negatively correlated with change in gait speed one year after TKA (r = −0.51, p = .007, Fig. 5), indicating a larger improvement in gait speed for those with lower baseline gait speeds. Visual inspection of the scatterplot suggested that this association was disproportionally influenced by one observation. To check the robustness of this association, we removed this observation, which lowered the correlation coefficient to −0.27 (p = .186).

**Table 3:** Changes in mobility metrics from baseline to one year and two years post-TKA.

	Baseline	1 year post-TKA – Baseline		2 years post-TKA – 1 year post-TKA	
	Mean [95%CI]	Mean difference [95%CI]	*p*-value	Mean difference [95%CI]	*p*-value
Gait speed – dominant peak (m/s)	1.15 [1.02, 1.27]	0.10 [0.04, 0.15]	<0.01	−0.00 [−0.06, 0.06]	0.93
Gait speed – 95th percentile (m/s)	1.35 [1.23, 1.48]	0.07 [0.03, 0.10]	<0.01	−0.01 [−0.05, 0.02]	0.43
Stride time – median (s)	1.17 [1.07, 1.26]	−0.03 [−0.06, −0.01]	0.01	−0.01 [−0.03, 0.02	0.59
Stride length – median (m)	1.27 [1.17, 1.38]	0.07 [0.02, 0.11]	<0.01	−0.00 [−0.05, 0.04]	0.95
Peak turning velocity (deg/s)	94.6 [90.8, 98.4]	1.9 [−1.5, 5.4]	0.28	0.3 [−3.9, 4.5]	0.88
Maximum bout length	342 [192, 492]	67 [−64, 195]	0.32	140 [−11, 290]	0.08
# Strides/ hour	156 [71, 241]	81 [24, 139]	0.01	−20 [−82, 42]	0.53
Number of people reaching gait speed threshold of 0,1 m/s (n)					
More than 0.1 m/s change		12	NA	NA	NA
Less than 0.1 m/s change		1	NA	NA	NA

Means and 95%CI are shown for baseline mobility metrics. For comparisons between baseline and one year post-TKA, and one year post-TKA and two years post-TKA, mean differences, 95%CI and *p*-values are reported. 95%CI = 95% confidence interval. TKA = total knee arthroplasty; NA = not available.

Maximum gait speed increased with 0.07 m/s (95%CI: [0.03, 0.10], *p* < 0.001) following TKA. Furthermore, stride time was lower (mean difference: 0.03 s; 95%CI: [−0.06, −0.01], *p* = 0.008) and stride length higher (mean difference: 0.07 m; 95%CI: [0.02, 0.11], *p* = 0.003) at one year post-TKA compared to baseline. The mean number of strides/h increased by 81 strides (95%CI: [24, 139], *p* = 0.008) from baseline to one year post-TKA. Turning velocity and maximum bout length did not change from baseline to one year post-TKA. Between one and two years post-TKA, no further changes in walking metrics were observed.

The KOOS-PS was 16 points lower (95%CI: [−22, −11]) one year post-TKA compared to baseline. NRS pain at rest decreased with 3 points (95%CI: [−4, −2]), and NRS pain during activity with 4 points (95%CI: [−5, −3]). No changes in patient-reported outcomes were observed between one year and two years post-TKA. Detailed results for patient-reported outcomes can be found in Appendix B.

## 4 Discussion

This study compared daily-life walking after TKA to healthy participants and evaluated the extent to which walking recovered up to two years post-TKA. At one year, minor differences in all walking metrics with healthy participants were observed. Compared to baseline assessment, gait speed increased after one year, but only 12 out of 27 participants exceeded the prespecified threshold of 0.10 m/s of change in gait speed. Individuals showed an increased number of strides per hour at one year post-TKA, reflecting increased walking activity, but did not show improvement in the duration of uninterrupted walking. The speed of turning did not change from baseline to post-TKA timepoints.

At one year post-TKA, patients demonstrated gait characteristics that were largely comparable to those of their healthy peers. In particular, the average walking speed of ∼1.25 m/s - although 0.10 m/s lower than the healthy group - indicates that most individuals were capable of walking at a pace which would not substantially limit daily life activities [24]. However, our results also suggest that one year post-TKA, patients may still experience difficulty with prolonged walking. One year after TKA, the patients had an average maximum gait bout length which was roughly half the time and distance covered on average by healthy individuals. Such reduced ability to walk longer distances could represent a significant limitation in daily life, particularly in contexts that require prolonged walking without rest.

When evaluating walking after TKA as a change from the preoperative status, we observed substantial heterogeneity in changes in gait speed, with about half of participants not improving beyond the threshold of 0.10 m/s. This contrasted with large and clinically meaningful reductions in pain scores [37,38] and improvements in self-reported physical function. In line with these findings, a recent meta-analysis showed that pain and mobility follow distinct recovery trajectories after TKA, differing in timing and magnitude of recovery, indicating that they are at least partly independent [39]. Together with previously reported discordance between (changes in) pain and in walking [11,40,41], these results raise the possibility that pain is not the primary factor limiting walking in daily life, even before TKA. In our group, gait speed of the TKA group was ∼1.2 m/s at baseline, which can be considered as mildly impaired when compared to values of unimpaired people and other disease populations with walking impairments [42,43]. This also raises the question whether people had enough room for improvement in daily-life walking before surgery. We tested this by associating baseline gait speed with the observed change at one year. However, this analysis did not provide convincing evidence that high preoperative walking performance constrained postoperative gains.

The turning outcomes in this study did not reveal a clear pattern of impairment or recovery of daily-life turning among individuals undergoing TKA. Pain, stiffness, or a sense of instability during rotational movements may contribute to more cautious, slower turning in people with knee OA. In previous work, we observed marked deficits in turning capacity during 180° turns in clinical assessments in people with TKA compared to healthy controls [11]. Although performance improved over time, it remained clearly below levels of the healthy group one year postoperatively [11]. In contrast, daily-life data showed only small preoperative differences between people with knee OA and healthy peers [3]. In the present longitudinal study, no meaningful improvement in daily-life turning was detected, and the small difference from healthy individuals persisted one year post-TKA. An important consideration is that, based on the peak turn velocity of different turn angles [3], 180° turns as used in clinical assessments comprised only a small fraction of all turns recorded in daily life. This suggests that turning difficulties in people with knee OA are more likely to emerge during sharp turns, whereas the more frequent, smaller turns typical in everyday ambulation may mask between-group differences when turning is recorded over a full week.

A number of limitations to our study should be considered when interpreting our results. First, to be able to attribute changes in walking primarily to TKA, and minimize effects from other joints, we excluded individuals with pain in joints other than the index knee. This came with the downside of limited generalizability, as OA in multiple joints is very common [44]. It might be expected that this excluded group would have even smaller improvements after surgery, due to persistent pain in other joints. Nonetheless, when comparing our study sample with the general TKA population in the Netherlands, our group was very similar in terms of demographics and patient-reported outcomes [45]. Furthermore, participants in this study received cruciate-retaining knee arthroplasty, which may hinder generalizability to the broader TKA population. However, previous research reported no differences in recovery of pain and function between conventional TKA and cruciate-retaining TKA [46], which is thought to translate to objective measures of walking. Secondly, differences in BMI were apparent between our TKA and healthy group, in line with the general TKA population. A healthy group matched on BMI apart from age and sex would have been a better comparison, although it must be noted that due to the fact that BMI is a strong risk factor for development of OA, finding this group is a challenging task. Another limitation is that we did not reach our intended sample size. We originally powered the study to have 29 participants at one year post-TKA. However, we had a higher dropout rate than foreseen (5 vs 3 foreseen), resulting in data of 27 participants analyzable for the between-group comparison at 1 year. This slightly smaller sample size may have affected the precision of our estimates. In addition, because no TKA-specific MCID for gait speed has been established, we set the threshold of meaningful improvement based on the range of MCIDs established in previous work in different groups of people with gait pathologies. For future studies, it would be recommended to evaluate whether this threshold is adequate for evaluation of TKA procedures. Finally, one should note that our assessment was geared toward capturing walking metrics rather than full-day physical activity tracking. Participants wore sensors only during daytime due to battery constraints, likely biasing data toward more active periods. As such, our walking activity metric of number of strides/hour should be interpreted with caution and cannot be compared directly to other studies using 24-h activity tracking.

In conclusion, individuals after TKA demonstrated only slight walking limitations compared to healthy peers when monitored over a full week, likely reflecting their already high preoperative functional status. Moreover, less than half of individuals reached a clinically relevant change in gait speed one year after surgery. As walking is the largest contributor to daily physical activity [47] and remains a top priority for individuals considering TKA [[4], [5], [6]], these results underscore the need to set realistic expectations regarding improvement of walking after surgery.

## Author contributions

Conception and design: R.J. Boekesteijn, K.C. Defoort, K. Smulders.

Analysis and interpretation of the data: F.J. Bruning, R.J. Boekesteijn, K. Smulders.

Drafting of the article: F.J. Bruning, R.J. Boekesteijn, K. Smulders.

Critical revision of the article for important intellectual content: F.J. Bruning, R.J. Boekesteijn, K.C. Defoort, S. Susan, C.H.M. van den Ende, K. Smulders.

Final approval of the article: F.J. Bruning, R.J. Boekesteijn, K.C. Defoort, S. Susan, C.H.M. van den Ende, K. Smulders.

Provision of study materials or patients: R.J. Boekesteijn, K.C. Defoort, S. Susan.

Obtaining of funding: K. Smulders, K.C. Defoort.

Administrative, technical or logistic support: S. Susan.

Collection and assembly of data: F.J. Bruning, R.J. Boekesteijn, S. Susan.

## Role of the funding source

Smith & Nephew Orthopaedics AG, Switzerland sponsored this study. The funders had no role in the design and conduct of this study.

## Conflict of interest

The authors have no conflicts of interest to declare.

## Figures and Tables

**Table B1:** Patient-reported outcome measures (PROMs) per time point, and mean differences + 95%-Confidence Intervals (95%CI) between time points.

	Baseline Mean (sd)	1 year post-TKA Mean (sd)	2 years post-TKA Mean (sd)	1 year post-TKA – baseline Mean difference [95%CI], *p*-value	2 year post-TKA – 1 year post-TKA Mean difference [95%CI], *p*-value
KOOS-PS (0–100)	45 (12)	29 (13)	29 (18)	−16 [−22, −11], *p* < 0.001	−0 [−6, 6], *p* = 0.997
NRS pain – rest (0−10)	4 (2)	1 (1)	1 (2)	−3 [−4, −2], *p* < 0.001	−0 [−1, 1], *p* = 0.862
NRS pain – activity (0−10)	6 (2)	2 (2)	2 (2)	−4 [−5, −3], *p* < 0.001	−0 [−1, 1], *p* = 0.839

PROMs are reported as mean (sd) per time point, and differences are reported as mean difference [95%CI] and *p*-value. KOOS-PS = Knee Injury and Osteoarthritis Outcome Score – Physical function Short form, NRS = Numeric Rating Scale.

**Fig. B1:**
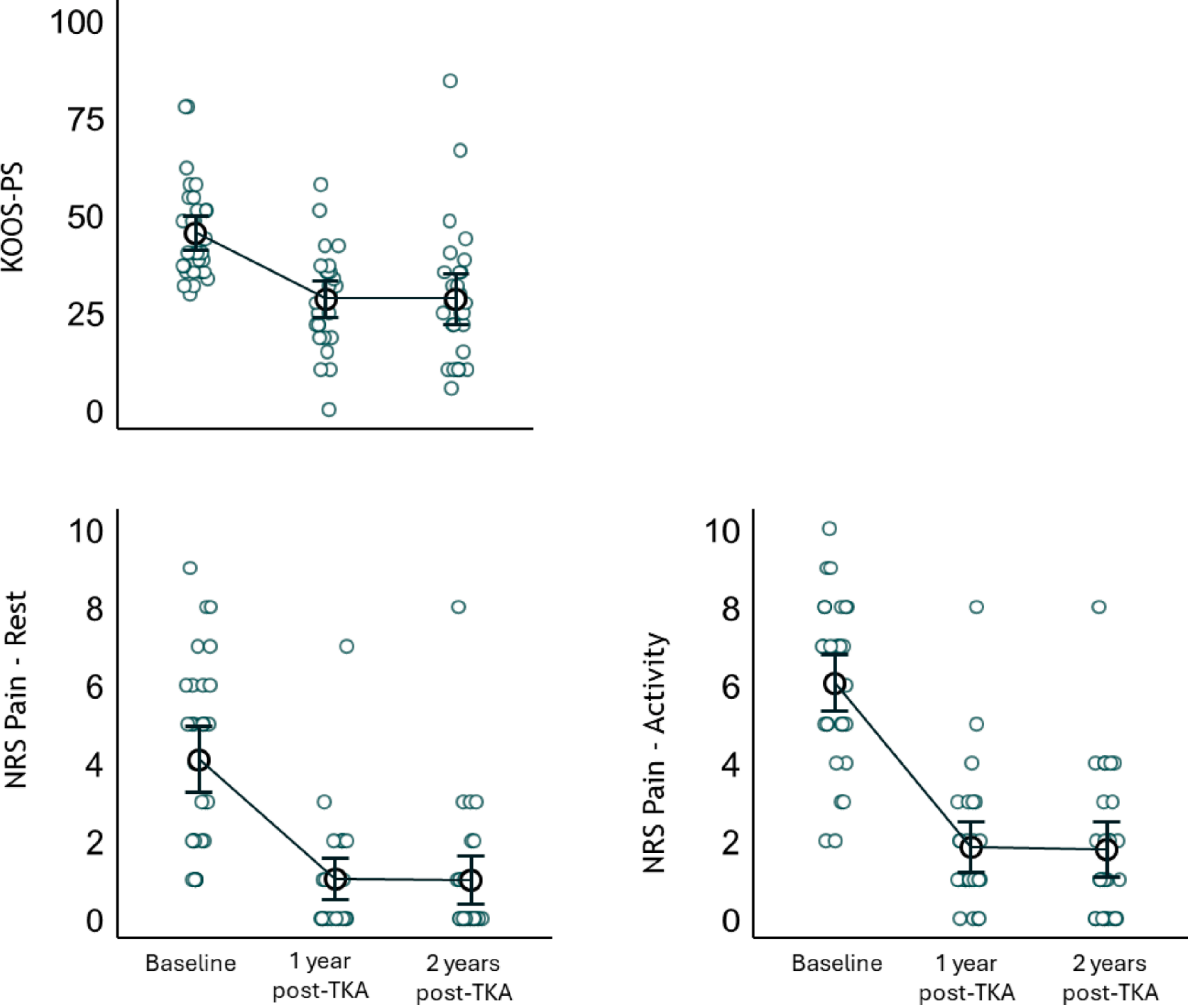
Outcomes for PROMs over time. Values are shown as mean + 95%CI, with dots representing individual values. KOOS-PS = Knee Injury and Osteoarthritis Outcome Score – Physical function Short form, NRS = Numeric Rating Scale. For NRS pain in rest and activity.

## Appendix A Inclusion and exclusion criteria

### Inclusion criteria for individuals undergoing TKA

- Non-inflammatory knee osteoarthritis, (Kellgren & Lawrence ≥ 2 on radiographic image of the knee).
- Scheduled for primary cemented total knee arthroplasty
- Age between 40 and 80 years on the day of surgery
- Stable health (ASA-score ≤3), and no cardiac, pulmonary, hematological or other conditions with high operative risks
- Correctable or <10° non-correctable varus or valgus deformity of the knee
- Able to give informed consent

### Exclusion criteria for individuals undergoing TKA

- Body mass index >35 kg/m^2^
- Previous major, non-arthroscopic surgery to the study knee, including high tibial osteotomy
- Active, local infection or systemic infection
- Prior high-energy trauma to the study knee or previous rupture of the posterior cruciate ligament
- Suspicion of posterior cruciate ligament rupture at clinical examination
- Incomplete or insufficient tissue surrounding the knee
- Severe damage to any knee ligament other than the anterior cruciate ligament
- Osteoporosis with active medical treatment
- Physical, emotional, neurological conditions that would compromise compliance with post-operative rehabilitation and follow-up
- Bone quality compromised by disease or infection leading to inability to support and fixate the prosthesis
- Fixed flexion deformity >10°
- Extension deficit >30°
- Improper functioning of patella tendon on the affected side
- Impaired extensor strength (MRC Grade <4)
- Previous patellectomy in the study knee
- Moderate to severe pain in the contralateral knee defined as an average score of >4 on items 3–6 of the Short Brief Pain Inventory
- Moderate to severe hip or ankle pain as defined as an average score of >4 on items 3–6 of the Short Brief Pain Inventory
- Previous or scheduled knee (in the next 12 months), hip or ankle replacement surgery
- Any musculoskeletal disease other than OA, neurological disease, or uncorrected visual impairment that affects gait or balance

### Inclusion criteria healthy controls

- Age between 40 and 80 years on the day of baseline measurement
- In stable health (ASA-score ≤3)
- Able to give informed consent
*Exclusion criteria healthy controls*
- Moderate to severe pain in the one or both knees defined as an average score of >4 on items 3–6 of the Short Brief Pain Inventory
- Moderate to severe hip or ankle pain as defined as an average score of >4 on items 3–6 of the Short Brief Pain Inventory
- Previous or scheduled (in the next 24 months), knee hip or ankle replacement surgery
- Any musculoskeletal, neurological or uncorrected visual impairments that impairs gait or balance.

## Appendix B Results PROMs

KOOS-PS, NRS pain in rest, and NRS pain in activity all showed improvements at one year post-TKA compared to baseline, but no further improvements on Patient-reported outcome measures were observed at two years post-TKA (Table B1, Figure B1).
