# Supplementary material for: Recovery of daily-life walking after total knee arthroplasty: A two-year longitudinal study and comparison with healthy controls
Source: Osteoarthr Cartil Open. 2026 Jun 4;8(3):100835. doi: 10.1016/j.ocarto.2026.100835 (PMC13312459; doi:10.1016/j.ocarto.2026.100835)
Supplement: Multimedia component 1 [file mmc1.docx]

Supplementary I – Association gait speed and gait bout length

Aim

The aim of this supplementary analysis was to determine how gait speed across different bout lengths differs between individuals one year after total knee arthroplasty (TKA) and healthy participants, and to investigate the influence of gait bout length on gait speed before TKA and one year after TKA.

Methods

*Data processing*

Gait bouts were categorized into six bins (i.e. 4-10, 11-20, 21-40, 41-80, 81-160 and 160+ strides). For each bin, the mean gait speed of all gait bouts was calculated per participant. For the comparison between one year post-TKA and healthy participants, only participants with data and their matched healthy participants were taken into analysis. For the comparison between baseline and one year after TKA, only participants who had data both at baseline and at one year post-TKA were used for analysis.

*Statistical analysis*

Independent t-tests were used to compare individuals at one year after TKA to healthy participants per gait bout bin. Within-subjects dependent t-tests were conducted per bin to assess differences between gait speed at baseline and one year after TKA.

Results

No significant differences on gait speed were found between individuals one year post-TKA and healthy participants for each gait bout bin (Figure 1A). At one year post-TKA, gait speed increased 0.08 m/s (95%CI: [0.04, 0.13) for bouts containing 41-80 strides, 0.08 m/s ([95%CI: [0.02, 0.14]) for 81-60 strides, and 0.08 m/s (95%CI: [0.00, 0.16]) for bouts longer than 160 strides (Figure 1B).


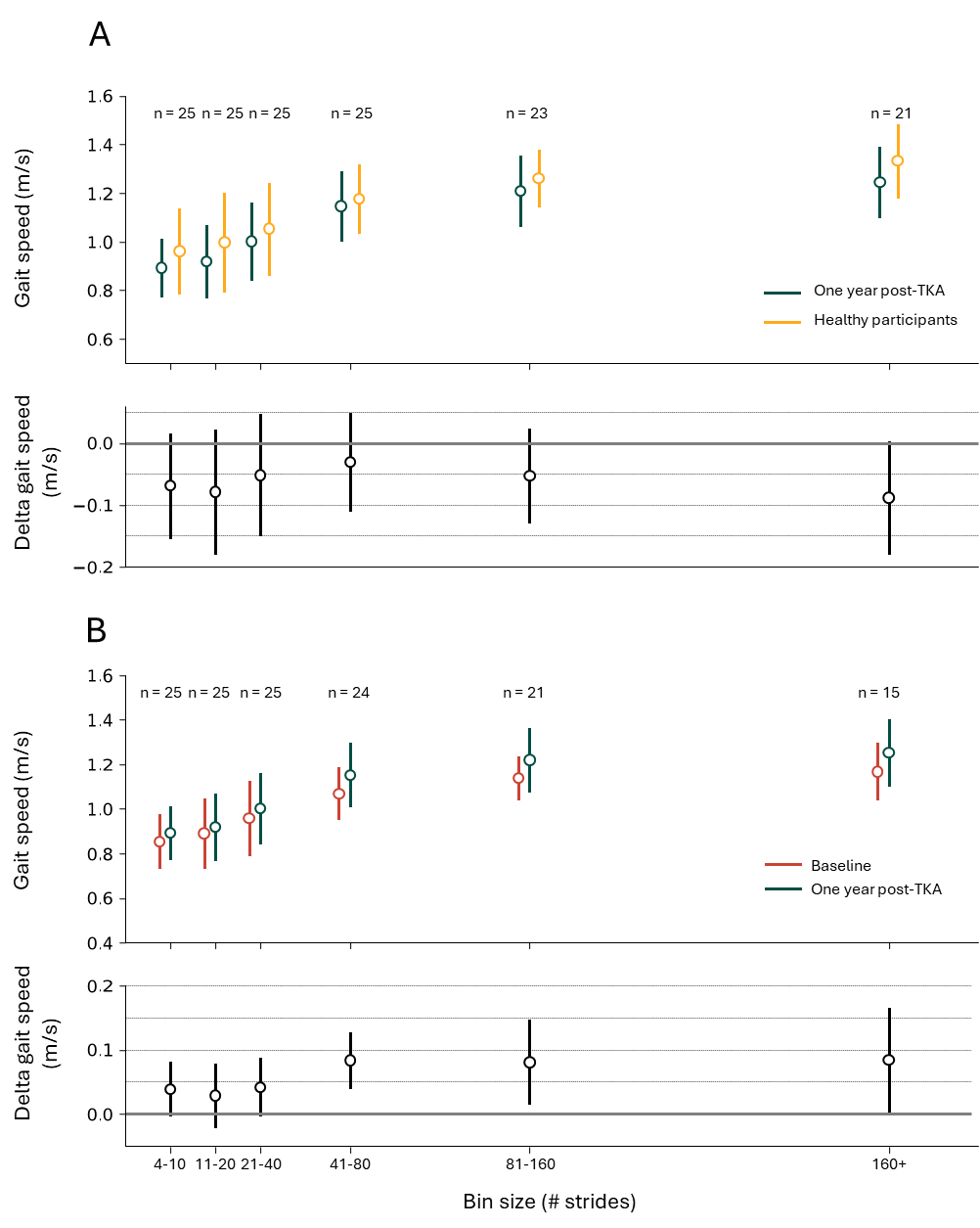


**Figure 1** – Effect of gait bout length on gait speed. Panel A shows the comparison between individuals one year post-TKA (green) and healthy participants (orange). Panel B shows the comparison for gait speed between one year post-TKA (green) and baseline (red). Numbers (n = ) above each comparison shows the number of individuals used in analysis per bin.
